# Design of a 0.5 V Chopper-Stabilized Differential Difference Amplifier for Analog Signal Processing Applications

**DOI:** 10.3390/s23249808

**Published:** 2023-12-13

**Authors:** Xinlan Fan, Feifan Gao, Pak Kwong Chan

**Affiliations:** School of Electrical and Electronic Engineering (EEE), Nanyang Technological University, Singapore 639798, Singapore; xfan006@e.ntu.edu.sg (X.F.); gaof0007@e.ntu.edu.sg (F.G.)

**Keywords:** differential difference amplifier, chopper stabilized, low voltage, damping factor, frequency compensation, sensor amplifier, analog signal processing

## Abstract

This paper presents a low-voltage low-power chopper-stabilized differential difference amplifier (DDA) realized using 40 nm CMOS technology. Operating with a supply voltage of 0.5 V, a three-stage DDA has been employed to achieve an open-loop gain of 89 dB, while consuming just 0.74 μW of power. The proposed DDA incorporates feed-forward frequency compensation and a Type II compensator to achieve pole-zero cancellation and damping factor control. The DDA has a unity-gain bandwidth (UGB) of 170 kHz, a phase margin (PM) of 63.98°, and a common-mode rejection ratio (CMRR) of up to 100 dB. This circuit can effectively drive a 50 pF capacitor in parallel with a 300 kΩ resistor. The use of the chopper stabilization technique effectively mitigates the offset and 1/f noise. The chopping frequency of the chopper modulator is 5 kHz. The input noise is 245 nV/sqrt (Hz) at 1 kHz, and the input-referred offset under Monte Carlo cases is only 0.26 mV. Such a low-voltage chopper-stabilized DDA will be very useful for analog signal processing applications. Compared to the reported chopper DDA counterparts, the proposed DDA is regarded as that with one of the lowest supply voltages. The proposed DDA has demonstrated its effectiveness in tradeoff design when dealing with multiple parameters pertaining to power consumption, noise, and bandwidth.

## 1. Introduction

In recent years, there has been a growing interest in the field of ultra-low-power and ultra-low-voltage circuit technologies [1,2,3,4,5]. This heightened interest can be attributed to advancements in technology and the rising demand for biomedical devices [6,7] and so forth. Circuits operating in the subthreshold region offer the advantage of significantly reduced power consumption, making them a practical approach for achieving both lower power consumption and lower supply voltages. Given the exponential relationship between V_GS_ and I_D_ of transistors operating in weak inversion, circuits can benefit from a high gm/I_D_ ratio, which enhances their performance. However, the limitation of low supply voltage poses challenges in the implementation of cascode amplifiers, thereby making the construction of high-gain amplifiers more complex. Thus, the need arises for multi-stage amplifiers and their stability through effective frequency compensation.

Research has indicated that considering the issues of reduced bandwidth and increased power consumption of multistage amplifiers, the three-stage amplifier is a popular topology for most low-voltage amplifier designs, which can provide a sufficient DC gain for most circuits [8]. In addition to the conventional three-stage nested Miller compensation (NMC), several advanced frequency compensation techniques have been developed. These include Multipath MNC (MNMC) [9], Nested Gm-C Compensation (NGCC) [10], and Damping-Factor-Controlled Frequency Compensation (DFCFC) [11]. These well-known topologies utilize pole-zero cancellation and damping factor control techniques to expand bandwidth and optimize the phase margin.

The differential difference amplifier (DDA) has been extensively employed in the biomedical field [12,13,14,15], in fully differential amplifiers [16], in filters [17], in data converters [18], in analog building blocks [19], in sensor signal-processing amplifiers [20], and so forth. It relies on the comparison of two floating input voltages in a high-gain amplifier circuit architecture. This relaxes the component count for processing floating analog signals. As such, less complexity is required. Therefore, it can achieve a high common-mode rejection ratio (CMRR) easily through its straightforward implementation and simplicity.

Similar to conventional CMOS operational amplifiers, DDAs also encounter limitations related to DC offset and low-frequency 1/f noise. These non-ideal effects can be mitigated using well-established dynamic offset cancellation methods. These methods commonly involve auto-zero techniques [21,22] and chopper stabilization techniques [23,24,25,26,27]. The chopper stabilization technique is a continuous-time design which can effectively reduce low-frequency noise and DC offset by modulating and demodulating signals in continuous time. It can achieve offset voltages of several microvolts and diminishes 1/f noise to over ten times lower than the original circuit without the chopper stabilization technique. However, the majority of chopper amplifiers that are designed will consume high power. This is mainly because a higher supply is needed to operate choppers, which are realized by MOS switch transistors in the triode region.

The application of the proposed DDA can be applied to ECG monitoring as an example. The ECG signals exhibit low amplitudes, ranging from 0.5 mV to 8 mV, necessitating an instrumentation amplifier as the analog frontend. The typical ECG signal amplitude is about 1 mV. Moreover, due to its susceptibility to noise, the application requires analog front-end circuits (AFE) for pre-amplification before processing the signals. Table 1 shows the typical specifications of analog front-end circuits, covering aspects such as bandwidth, noise, CMRR, and PSRR.

This paper presents a new chopper-stabilized DDA with a supply voltage of 0.5 V. This amplifier effectively addresses the challenge of achieving a high open-loop gain in low-voltage applications while simultaneously reducing power consumption. The key contribution of this paper lies in the novel frequency compensation technique employed for the three-stage chopper-stabilized DDA, whilst providing quantitative results of reducing DC offset and noise through the application of the low-voltage chopper technique. The proposed circuit combines the feed-forward compensation technique and a type II compensator to achieve sufficient bandwidth and robust stability. The amplifier has demonstrated excellent robustness, CMRR, and noise suppression performance, making it suitable for a wide range of analog signal processing applications which include bioelectrical signal processing, filter implementation, analog circuits, sensor interfaces, and so forth.

The following section of this paper is organized as follows. Section 2 reviews the previous design of the low-voltage DDA. Section 3 describes the design of the proposed chopper-stabilized DDA. Section 4 presents the simulation results and discussion. Section 5 summarizes the concluding remarks.

## 2. Review of Low-Voltage DDA Design

**Figure 1:**
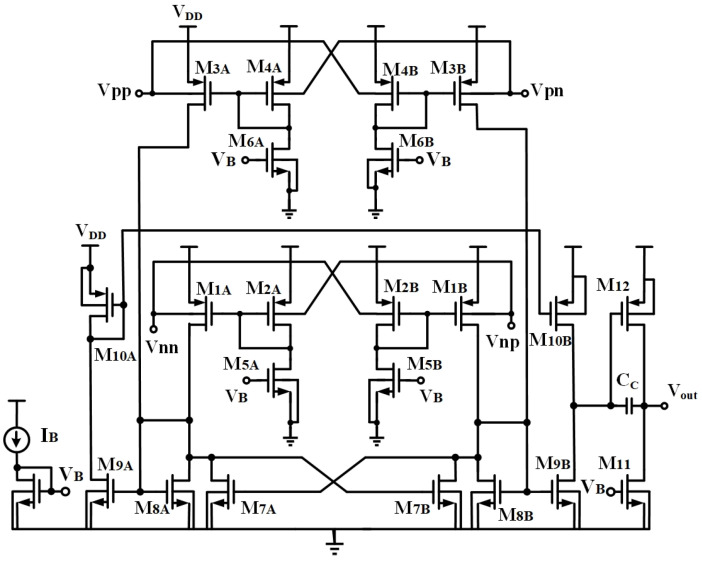
Non-tailed bulk-driven DDA.

Low supply voltages can significantly restrict the input common-mode voltage range. To tackle this challenge, bulk-driven amplifiers [28,29,30] employ bulk-driven input transistors operating in the subthreshold region. Additionally, the conventional tail current source in the input differential amplifiers can be eliminated. This modification expands the common-mode input voltage range and reduces the V_DD_/V_TH_ ratio. The detailed circuit is depicted in Figure 1.

Due to the bulk-driven topology, the circuit allows for a rail-to-rail common-mode input swing. However, the application of the bulk-driven technique will greatly minimize the transconductance of the input stage, which leads to higher noise and also limits the bandwidth. Thus, most circuits are constrained to a gain bandwidth of only a few tens of kilohertz and exhibits relatively high noise levels.

**Figure 2:**
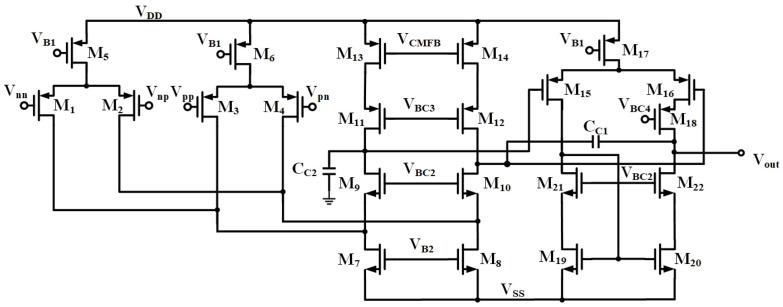
DDA with a fully differential folded cascode input stage and differential-to-single-ended converter as the output stage (common-mode feedback circuit not shown).

In addition, the DDA can be designed to operate in the subthreshold region in order to lower the operating supply voltage. A gate-source-driven DDA, which is able to operate at a supply voltage of 1 V, is shown in Figure 2 [31]. The first stage consists of transistors M_1_–M_14_, which not only implements the inputs of the DDA but also employs a fully differential folded cascode structure to enhance the gain as the first step. The signal from the first stage is then converted to a differential-to-single-ended gain stage consisting of transistors M_15_–M_22_ to further boost the gain. It is noted that M_18_ is added at the output branch to form the cascode structure to increase the gain as the second step. C_C1_ is the Miller frequency compensation capacitor, whereas the grounded C_C2_ is used to stabilize the common-mode feedback loop in the differential amplifier.

Due to the larger transconductance gm of the gate–source bias transistor, the gate–source-driven amplifier is able to achieve lower noise than the bulk-driven technique. The use of a folded cascode structure plus differential-to-single-ended converter enables the DDA to achieve an overall open-loop gain exceeding 100 dB. However, it imposes limitations on the output swing and input common-mode range. This configuration proves challenging to operate with a supply voltage as low as 0.5 V.

## 3. Proposed Low-Voltage Chopper-Stabilized DDA Design

### 3.1. Chopper-Stabilized DDA Operation Principle

**Figure 3:**
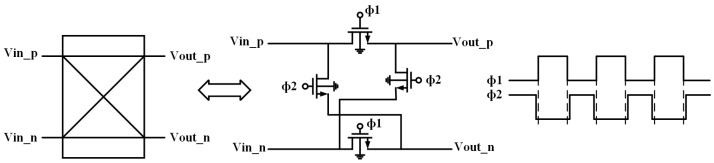
Chopper modulator.

Figure 3 depicts the chopper modulator employing NMOS transistors as switches. The expressions for the time-domain and Fourier transformation of the chopper modulator with a chopping frequency of $f_{chopper}$ are given as follows:

(1) $$m\left(t\right)=\left\{\begin{array}{c} 1,\;\;\;\;\;0<t<\frac{T_{chopper}}{2}\\ -1,\;\frac{T_{chopper}}{2}<t<T_{chopper} \end{array}\right.,\;T_{chopper}=\frac{1}{f_{chopper}}$$

(2) $$\begin{array}{c} m\left(t\right)=\sum_{k=1}^{\infty }\frac{4}{k\pi }\sin\left(2\pi kf_{\mathrm{chopper}\;}t\right) \end{array},\;k\;is\;odd$$

**Figure 4:**
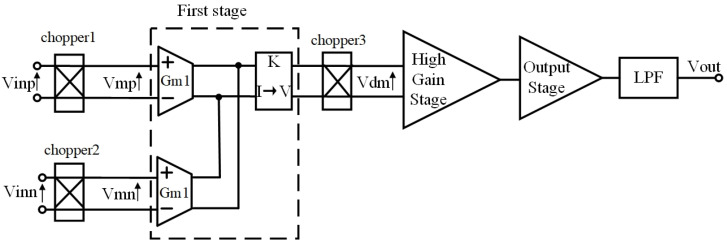
The block diagram of the proposed chopper-stabilized DDA.

From (2), it can be seen that the frequency domain characteristic of the chopper modulator involves harmonics exclusively of odd order, with the absence of a DC component. Figure 4 illustrates the block diagram of the proposed chopper-stabilized DDA circuit. Two pairs of input differential voltage signals are simultaneously modulated by the chopper and subsequently combined with noise and DC offset voltage at the amplifier inputs. These combined signals are then directed into the first stage. The signals at the transconductance inputs of the first stage amplifier can be expressed as follows:

(3) $$V_{\mathrm{mp}}\left(t\right)=V_{\mathrm{inp}\;}\cdot m\left(t\right)+V_{OSp}+V_{Np}\;\;=V_{inp}\sum_{k=1}^{\infty }\frac{4}{k\pi }\sin\left(2\pi kf_{chopper}t\right)+V_{OSp}+V_{Np},\;k\;is\;odd$$

(4) $$V_{\mathrm{mn}}\left(t\right)=V_{\mathrm{inn}\;}\cdot m\left(t\right)+V_{OSn}+V_{Nn}\;\;=V_{inn}\sum_{k=1}^{\infty }\frac{4}{k\pi }\sin\left(2\pi kf_{chopper}t\right)+V_{OSn}+V_{Nn},\;k\;is\;odd$$

where $V_{OSp}$ and $V_{OSn}$ represent the offset voltage of each input port, while $V_{Np}$ and $V_{Nn}$ denote the noise of each input port. Equations (3) and (4) reveal that the input choppers shift the differential input signals to the odd harmonics of the chopping frequency, while noise and bias voltages remain in the baseband spectrum. Subsequently, the input combined signals are converted into current signals by transconductors with identical transconductance gains of $G_{m1}$. At the outputs of transconductors, these currents are summed and then transformed into voltage signals by a transimpedance amplifier with a gain denoted as K. The output voltage signal of the transimpedance amplifier undergoes further processing through the output chopper demodulator. The resulting voltage signal can be expressed as

(5) $$V_{dm}\left(t\right)=K\cdot m\left(t\right)\cdot \left[G_{m1}{\cdot V}_{mp}\left(t\right)-G_{m1}\cdot V_{mn}\left(t\right)\right]\;\;={KG}_{m1}\left(V_{\mathrm{inp}\;}-V_{inn}\right)\left(\sum_{k=1}^{\infty }\frac{4}{k\pi }\sin\left(2\pi {kf}_{\mathrm{chopper}\;}t\right)\;\right)\left(\sum_{j=1}^{\infty }\frac{4}{j\pi }\sin\left(2\pi {jf}_{\mathrm{chopper}\;}t\right)\right)\;\;+{KG}_{m1}\left(V_{\mathrm{OSp}\;}-V_{OSn}+V_{Np}-V_{Nn}\right)\left(\sum_{j=1}^{\infty }\frac{4}{j\pi }\sin\left(2\pi {jf}_{\mathrm{chopper}\;}t\right)\;\right),\;k,j\;is\;odd$$

The first term of the above equation is the result of two modulations of the input signal, and its expansion is obtained as

(6) $$\begin{array}{c} KG_{m1}(V_{inp}-V_{inn})\left(\sum_{k=1}^{\infty }\frac{4}{k\pi }\sin\left(2\pi kf_{\mathrm{chopper}\;}t\right)\right)\left(\sum_{j=1}^{\infty }\frac{4}{j\pi }\sin\left(2\pi jf_{\mathrm{chopper}\;}t\right)\right)\\ \;=\left\{\begin{array}{c} KG_{m1}\left(V_{inp}-V_{inn}\right)\frac{8}{\pi^{2}}-KG_{m1}\left(V_{inp}-V_{inn}\right)\frac{8}{\pi^{2}}cos\left(4\pi f_{chopper}t\right),\;k=j=1\\ KG_{m1}\left(V_{inp}-V_{inn}\right)\sum_{k=1,j=1}^{\infty }\;\frac{4}{k\pi }\frac{4}{j\pi }\left(\frac{\cos\left(k-j\right)2\pi f_{\mathrm{chopper}\;}t}{2}-\frac{\cos\left(k+j\right)2\pi f_{\mathrm{chopper}\;}t}{2}\right),\;others \end{array}\right. \end{array}$$

From (5) and (6), it can be observed that the demodulation process preserves the low-frequency component of the signal. Moreover, any offset voltage as well as the 1/f noise are primarily present at the odd harmonics of the chopping frequency. By implementing a low-pass filter, it becomes possible to effectively eliminate both the offset voltage and the low-frequency noise, thereby enhancing the overall signal quality. As the output chopper is placed before the high-gain stage, it does not suppress the noise from the high-gain stage and the buffer stage. Therefore, the gain of the first stage $A_{1}=K\cdot G_{m1}$ should be designed as high as possible to reduce noise. In addition, there exists a tradeoff in the design of the front-end stage. Increasing $G_{m1}$ comes at the cost of a reduced-input common-mode range.

### 3.2. Circuit Topology

**Figure 5:**
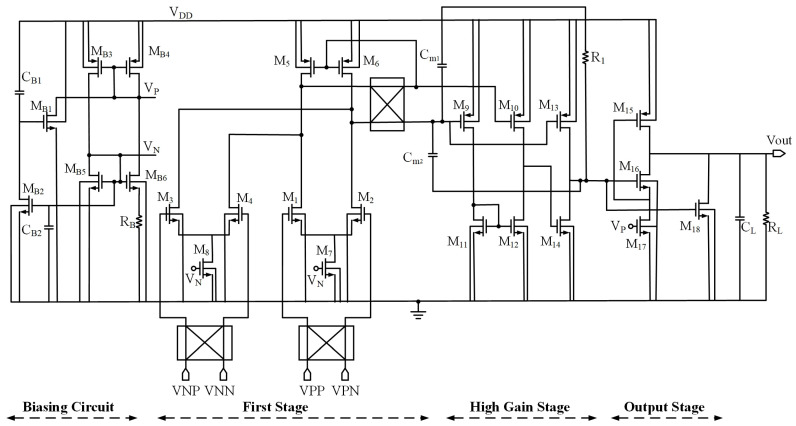
Circuit diagram of proposed chopper-stabilized DDA.

The circuit diagram of the proposed chopper-stabilized DDA is shown in Figure 5, and the sizes and types of components of the proposed circuit are shown in Table 2. The circuit consists of four stages, namely the biasing circuit, first stage, high-gain stage, and output stage. The biasing circuit employs a fast start-up bias circuit comprising transistors M_B1_ to M_B6_, capacitors C_B1_ and C_B2_, and resistor R_B_. The effective reduction in power consumption is achieved through the utilization of a capacitor startup network. In the first stage, both input ports of the circuit employ NMOS transistors, enhancing the matching performance between these two ports. The sizes of input transistors are the same and intentionally set relatively large to minimize flicker noise. Additionally, a current mirror load is employed to achieve improved power efficiency. The high-gain stage comprises a non-inverting amplifier using a current mirror load and an inverting amplifier with a current source load to enhance the total gain of circuit. Moreover, two feed-forward paths are implemented using transistors M_10_ and M_13_. Together with a type II compensator, comprising compensation capacitors C_m1_ and C_m2_, as well as a nulling resistor R_m_, the configuration forms a frequency compensation network, ensuring robust stability. The output stage employs an output buffer structure, consisting of a native NMOS transistor M_16_, along with two low-threshold voltage MOS transistors, M_15_ and M_17_, and a high-threshold voltage NMOS transistor M_18_. By combining two amplifiers to operate at different input signal ranges, the buffer permits rail-to-rail operation under a very low supply voltage. In addition, it offers a resistive driving capability due to the feedback transistor M_15_ that helps the reduction in output resistance of the buffer.

It is noted that the offset in the chopper-stabilized DDA comes from a mismatch in impedance between ports [26]. In addition, noise is partitioned into thermal or flicker noise components. Through chopper operation, the majority of the flicker noise component is reduced; hence, noise is dominated by thermal noise, which is related to g_m_ and I_d_ bias in the front-stage design. Stability is related to unity-gain bandwidth at a given power and capacitive load indirectly. It is directly related to the effectiveness of the frequency compensation technique as well as the architecture. In this paper, the power/bandwidth metric is adopted to quantify the stability because an amplifier will be subject to the stability issue when the bandwidth is enlarged at a given power and specific load.

**Figure 6:**
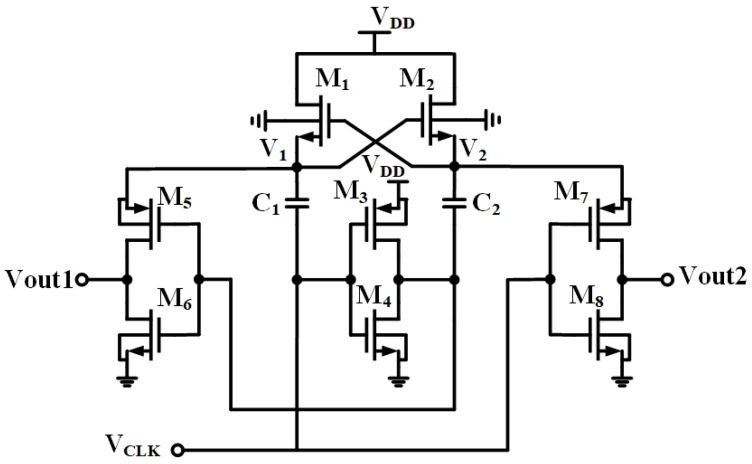
The clock booster for the proposed chopper-stabilized DDA.

For the low-voltage chopper modulator design, the clock generated by the oscillator is not sufficient to drive the chopper switches, due to the supply voltage limitation. Therefore, a clock booster is essential to enable the chopper to operate effectively under the low-voltage condition. The conventional Dickson charge pump circuit can achieve the multiplication of clock swing. However, its performance is constrained by the threshold voltage drop of NMOS transistors and the reverse charge-sharing phenomenon. In view of the previously mentioned issues, an alternative charge pump circuit [32] was employed, and its circuit is illustrated in Figure 6.

The amplitude of the input clock voltage to this circuit oscillates between 0 and V_DD_. When the clock voltage is high (V_DD_), the NMOS transistor M_2_ is turned on. Consequently, capacitor C_2_ starts being charged through the path from V_DD_ to transistor M_2_, gradually reaching a voltage level of V_DD_ (assuming negligible threshold voltage drop). As the clock transitions to a low state (0V), transistor M_3_ is switched on. This causes the voltage on the bottom plate of capacitor C_2_ to rise from 0 V to V_DD_. Simultaneously, the voltage on the top plate of capacitor C_2_ changes from V_DD_ to 2V_DD_ because of the charge accumulated by capacitor C_2_ during the preceding clock cycle. Consequently, V_2_ undergoes a transition from V_DD_ to 2V_DD_ while the clock is in the low state. The CMOS inverters composed of M_7_ and M_8_ are in control of V_CLK_. When the clock signal is high, transistor M_8_ is turned on, causing the output voltage V_out2_ to discharge to 0 V. Conversely, when the clock signal is low, transistor M_7_ is turned on. Since its source voltage is V_2_, the output voltage V_out2_ is pulled up to 2V_DD_. Therefore, the value of V_out2_ swings between 0 V and 2V_DD_, driven by the clock signal.

**Figure 7:**
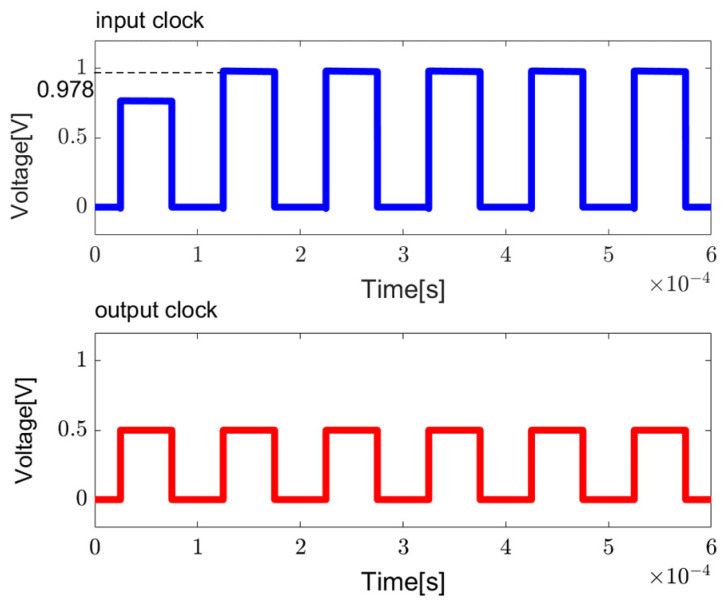
The simulation results for clock booster.

The output on the left side of the circuit V_out1_ operates in a similar principle but with opposite polarity. The clock signals V_out1_ and V_out2_ are complementary. Given that V_out1_ is in phase with the input clock, it is used as the final output. To minimize chip area, the capacitance on the right side is reduced. The sizes of components and simulation results are illustrated in Table 3 and Figure 7, respectively. From Figure 7, it is evident that the amplitude of the output signal can be boosted to almost twice the amplitude of the input clock, specifically increasing from 500 mV to 978 mV.

### 3.3. Frequency Compensation of Proposed DDA

#### 3.3.1. Transfer Function

**Figure 8:**
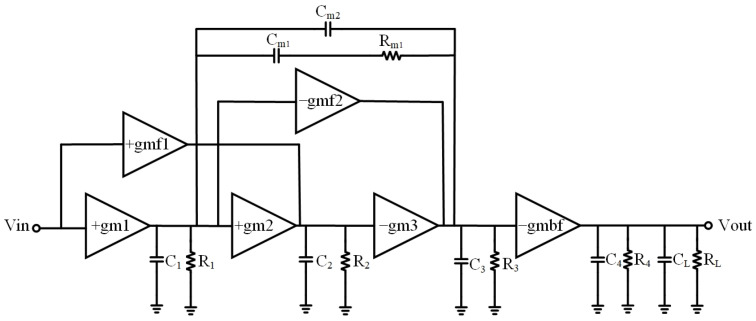
Frequency topology of proposed DDA.

**Figure 9:**
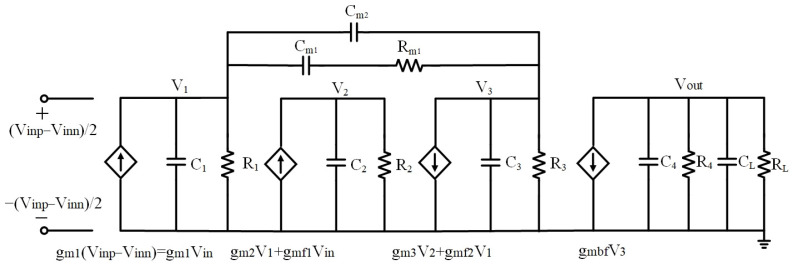
Small-signal model of proposed DDA.

The block diagram and small-signal diagram of the proposed DDA are illustrated in Figure 8 and Figure 9, respectively. The utilization of two components, namely (1) two feedforward transconductances and (2) a Type II compensator [33], impacts the frequency response. The role of the feedforward transconductances is to control the position of zeros. The addition of the Type II compensator serves two purposes. It facilitates the pole-zero cancellation by introducing a nulling resistor and simultaneously regulating the damping factor of the system related to the positions of the non-dominant complex poles.

Both feedforward transconductances are single transistors with transconductance $g_{mf1}$ and $-g_{mf2}$, respectively. The input signal for the first feedforward transconductance is from the input ports, and its output signal is connected to the input of the third stage. Meanwhile, the input signal for the second feedforward transconductance is from the output of the first stage, and its output signal is linked to the input of the buffer stage. The type II compensator consists of two capacitors $C_{m1}$ and $C_{m2}$ and a resistor $R_{m1}$, shown in Figure 8.

In order to analyze the stability of the circuit, the transfer function is first analyzed on the basis of the following assumptions. (1) $C_{L}\gg C_{m1},C_{m2}$. (2) Some parasitic capacitances, including $C_{1}\;and\;C_{2}$, are ignored due to small values. (3) The gain of each stage is much greater than one (i.e., $g_{mi}R_{i}\gg 1$). Thus, the transfer function is given as

(7) $$\begin{array}{c} \begin{array}{c} A_{v}\left(s\right)=\frac{A_{dc}\left(1+R_{m1}C_{m1}s+\frac{R_{m1}C_{m1}C_{m2}g_{mf}}{g_{m1}g_{m2}}s^{2}\right)}{\left(1+p_{1}s\right)\left(1+p_{2}s\right)\left(1+\frac{R_{m1}C_{m1}C_{m2}}{C_{m1}+C_{m2}}s+\frac{R_{m1}C_{m1}C_{m2}C_{3}}{g_{m2}g_{m3}R_{2}\left(C_{m1}+C_{m2}\right)}s^{2}\right)} \end{array} \end{array}$$

where $A_{dc}=g_{m1}g_{m2}g_{m3}g_{mbf}R_{1}R_{2}R_{3}R_{out}$, $p_{1}=-1/R_{1}R_{2}R_{3}g_{m2}g_{m3}\left(C_{m1}+C_{m2}\right)$ is the dominant pole, and ${\;p}_{2}=-1/(R_{4}|\left|R_{L}\right)C_{L}$ is a non-dominant pole. The symbols have their usual meanings. Thus, the gain bandwidth product is as follows:

(8) $$GBW=A_{dc}\cdot p_{1}=\frac{g_{m1}}{C_{m1}+C_{m2}}g_{mbf}R_{\mathrm{out}\;}$$

From (8), it is obvious that the gain bandwidth product is controlled by the compensation capacitors $C_{m1}$ and ${\;C}_{m2}$. In addition, there are two zeros which can be used to cancel the non-dominant pole $p_{2}$. Moreover, the damping factor and the position of the other two non-dominant poles $p_{3,4}$ can be optimized by controlling a combination of $R_{m1},\;C_{m1},\;and\;C_{m2}.$

#### 3.3.2. Stability Analysis

The position of two zeros from (7) can be obtained as

(9) $$z_{1,2}=\frac{-1\pm \sqrt{1-\frac{4g_{mf1}C_{m2}}{g_{m1}g_{m2}R_{m1}C_{m1}}}}{2R_{m1}C_{m1}}$$

It can be seen from (9) that the position of two zeros can be controlled by $g_{mf1}$. By setting ${\;g}_{mf1}\ll g_{m1}g_{m2}R_{m1}C_{m1}/4C_{m2}$, two zeros can be located on the real axis in the left half-plane. By choosing

(10) $$g_{mf1}=0.1\frac{g_{m1}g_{m2}R_{m1}C_{m1}}{4C_{m2}}$$

and in order to cancel $p_{2}$, the dimension condition of $C_{m1}$ is

(11) $$C_{m1}=\frac{1}{2}\frac{R_{out}}{R_{m1}}C_{L}$$

where $R_{out}=R_{4}||R_{L}$. In the proposed DDA circuit, $R_{out}$ can be one-tenth the magnitude of $R_{m1}$ due to a relatively small load resistor to be driven. Thus, a small dimension of $C_{m1}$ is achievable.

At this juncture, the expression for the system’s loop gain can be obtained as

(12) $$A_{v}\left(s\right)=\frac{A_{dc}\left(1+\frac{s}{z_{1}}\right)\left(1+\frac{s}{z_{2}}\right)}{\left(1+\frac{s}{p_{1}}\right)\left(1+\frac{s}{p_{2}}\right)\left[1+\frac{R_{m1}C_{m1}C_{m2}}{C_{m1}+C_{m2}}s+\frac{R_{m1}C_{m1}C_{m2}C_{3}}{g_{m2}g_{m3}R_{2}\left(C_{m1}+C_{m2}\right)}s^{2}\right]}$$

To facilitate the optimization of the loop for a balanced tradeoff between stability and transient characteristics, a careful arrangement of the locations of the other two poles $p_{3,4}$ is essential. The quadratic polynomial part of the denominator of the loop gain is written as

(13) $$H\left(s\right)=1+\frac{R_{m1}C_{m1}C_{m2}}{C_{m1}+C_{m2}}s+\frac{R_{m1}C_{m1}C_{m2}C_{3}}{g_{m2}g_{m3}R_{2}\left(C_{m1}+C_{m2}\right)}s^{2}$$

According to the basic theory of damping factor control, a second-order system exhibits the following characteristic equation:

(14) $$F\left(s\right)=1+s\left(2\zeta \right)\left(\frac{1}{\omega_{n}}\right)+s^{2}{\left(\frac{1}{\omega_{n}}\right)}^{2}$$

where ξ represents the damping factor, and $\omega_{n}$ is the natural frequency. By applying the above model to the proposed DDA, the expression of ξ and $\omega_{n}$ can be derived as

(15) $$\omega_{n}=\sqrt{\frac{g_{m2}g_{m3}R_{2}\left(C_{m1}+C_{m2}\right)}{R_{m1}C_{m1}C_{m2}C_{3}}}$$

(16) $$\zeta =\frac{1}{2}\frac{R_{m1}C_{m1}C_{m2}}{C_{m1}{+C}_{m2}}\sqrt{\frac{g_{m2}g_{m3}R_{2}\left(C_{m1}+C_{m2}\right)}{R_{m1}C_{m1}C_{m2}C_{3}}}$$

Based on the principle of damping factor control, when $\zeta =1/\sqrt{2}$, the system’s transition time is shorter than that of the critically damped case, and the oscillation amplitude is reduced. Thus, the circuit will gain better stability. From this, the expression for the first condition is given by

(17) $$\frac{R_{m1}C_{m1}C_{m2}}{\left(C_{m1}+C_{m2}\right)}=\frac{2C_{3}}{g_{m2}g_{m3}R_{2}}$$

The position of the non-dominant complex poles $p_{3,4}$ can be obtained from Equation (14), which gives

(18) $$\left|p_{3,4}\right|=\omega_{n}$$

and the phase margin *PM* of the system is

(19) $$PM=180^{\circ }-\tan^{-1}\left(\frac{GBW}{p_{1}}\right)-\tan^{-1}\left[\frac{2\zeta \left(\frac{GBW}{\left|p_{3,4}\right|}\right)}{1-{\left(\frac{GBW}{\left|p_{3,4}\right|}\right)}^{2}}\right]$$

In the above equation, the phase shift generated by the dominant pole is 90°, and if the phase shift generated by the second dominant pole is 30°, the total phase margin of the system is 60°. At this point, the system is stable and has the best transient response characteristic. In order to achieve this, it is required that

(20) $$\left|p_{3,4}\right|=2\sqrt{2}GBW$$

By combining (20), (18), (17), (15), and (11) with (8), the dimension condition of $C_{m2}$ and $R_{m1}$ can be derived as follows:

(21) $$C_{m2}=\frac{16g_{m1}g_{mbf}C_{3}}{g_{m2}^{2}g_{m3}^{2}R_{2}^{2}C_{L}}C_{3}$$

(22) $$R_{m1}=\frac{g_{m2}g_{m3}C_{L}}{8g_{m1}g_{mbf}C_{3}}R_{2}$$

By substituting (22), (21), and (11) into (10), the condition of $g_{mf1}$ can be obtained as

(23) $$g_{mf1}=\frac{{R_{2}^{3}R}_{out}C_{L}^{3}g_{m2}^{3}g_{m3}^{3}}{10,240R_{m1}C_{3}^{3}g_{m1}g_{mbf}^{2}}g_{m2}$$

Based on the above analysis, it becomes evident that it is possible to achieve small dimensions of the compensation capacitors $C_{m1}$ and $C_{m2}$ while maintaining good phase margins.

## 4. Simulation Results and Discussions

**Figure 10:**
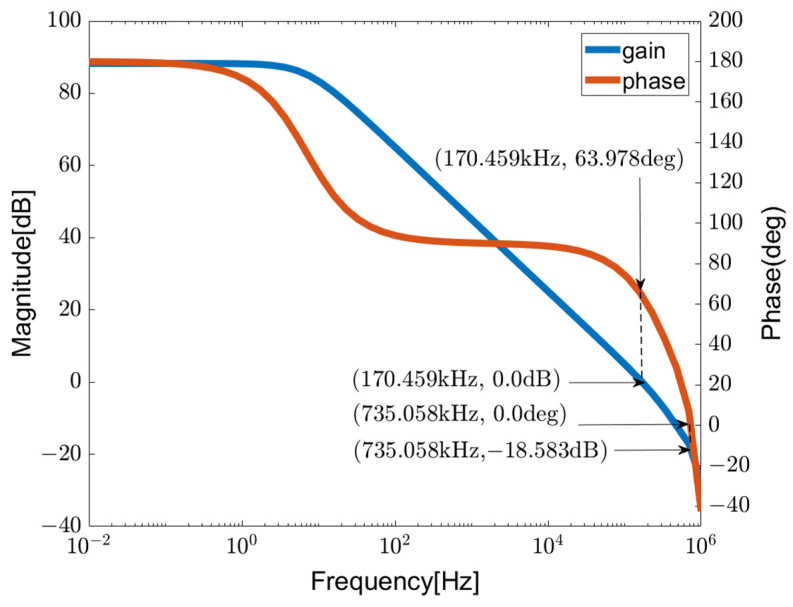
Open-loop gain and phase of chopper-stabilized DDA.

The proposed chopper-stabilized DDA is simulated using 40 nm CMOS technology, with a low supply voltage of 0.5 V. The simulation was analyzed using Cadence SpectreRF IC6.1.8-64b.500.1. The simulation results of the frequency response for the open-loop gain and phase of the chopper-stabilized DDA with a 5 kHz chopper frequency are depicted in Figure 10. With a 50 pF load capacitance and a parallel load resistor of 300 kΩ, the low-frequency gain reaches 89 dB, the unity-gain bandwidth is 170.5 kHz, and the phase margin is 63.98°. This verifies that the proposed frequency compensation is effective at ensuring stability in multi-stage amplifier design. Finally, it can be found that the voltage gain achieved surpasses the performance of the majority of sub-0.5 V designs.

**Figure 11:**
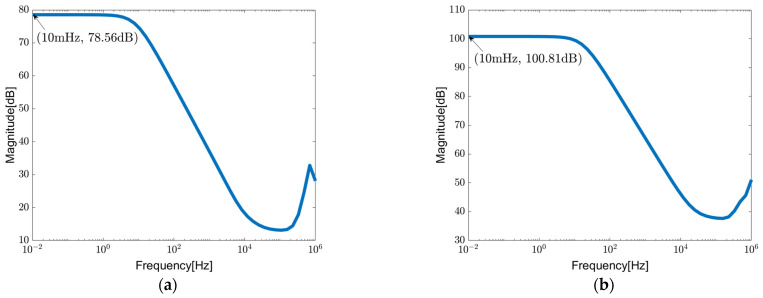
PSRR and CMRR of chopper-stabilized DDA in unity-gain configuration: (**a**) PSRR; (**b**) CMRR.

**Figure 12:**
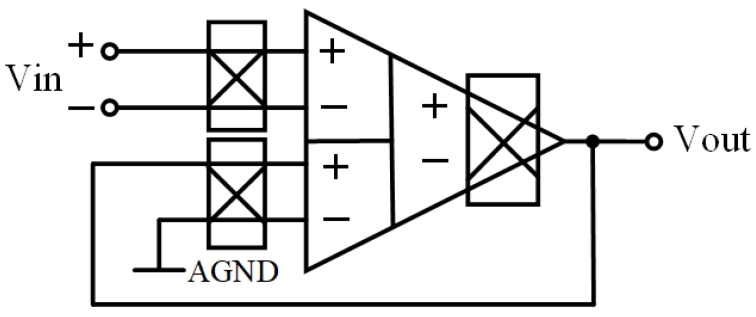
The unity-gain configuration of chopper-stabilized DDA.

Figure 11 shows the simulation results of the PSRR and CMRR of the proposed circuit in a unity-gain configuration, which is shown in Figure 12. It is worth noting that both parameters achieve comparably high values, owing to the unique structural characteristics of the DDA.

**Figure 13:**
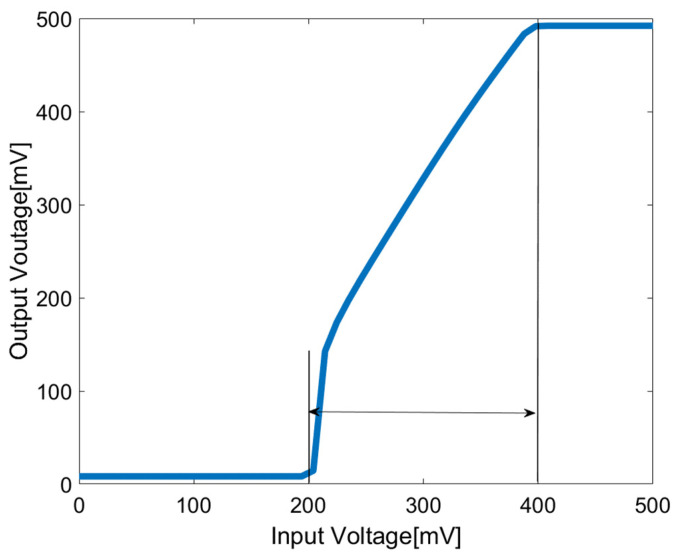
Common-mode range of the chopper-stabilized DDA in unity-gain configuration.

Figure 13 illustrates the input common-mode range and output common-mode range of the chopper-stabilized DDA. It can be observed that the input range with unity gain is 200 mV to 400 mV. Although the input common-mode range may not be extensive, this tradeoff is made to significantly enhance the overall gain and noise suppression performance of the proposed chopper DDA.

**Figure 14:**
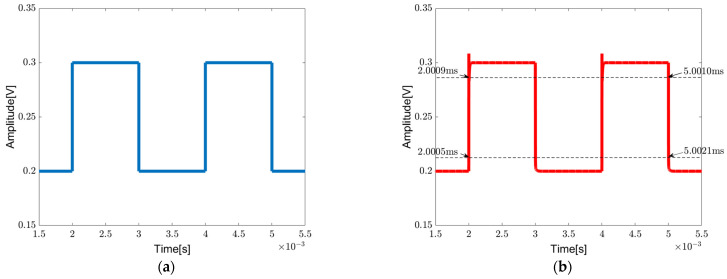
Transient pulse-wave response of the chopper-stabilized DDA in unity-gain configuration: (**a**) input, (**b**) output.

Figure 14a shows a 500 Hz pulse signal swinging from 200 mV to 300 mV being used for evaluation of the transient response of the chopper-stabilized DDA operating in a unity-gain configuration. The output signal is shown in Figure 14b, from which the slew rate SR+/SR− can be calculated as 150.00/54.54 V/ms, respectively.

**Figure 15:**
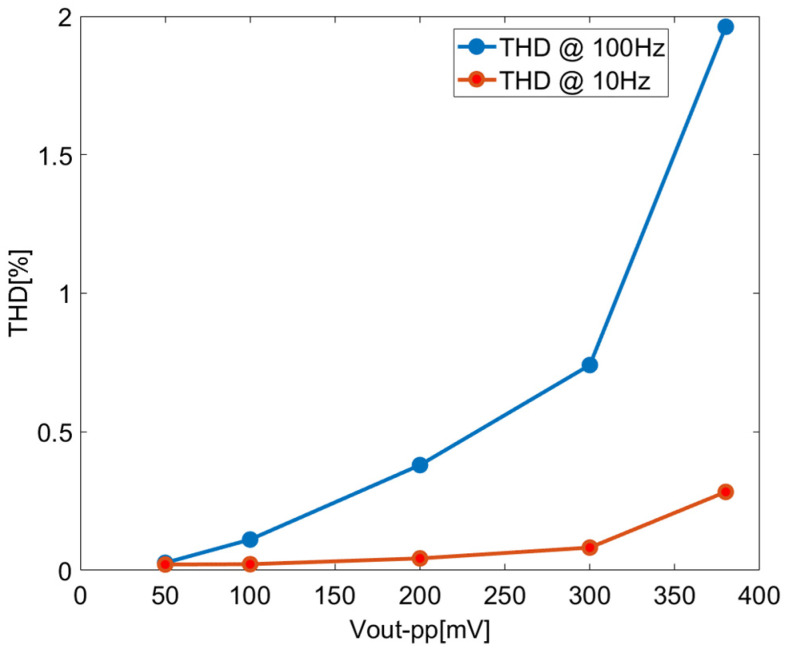
THD of closed-loop chopper DDA with unity-gain configuration.

Figure 15 illustrates the variation in total harmonic distortion (THD) with the amplitude of the output signal for 10 Hz and 100 Hz input signals. It shows that THD < 2% when the amplitude of the output signal is less than 380 mV pp. The output noise floor of the unity-gain configuration is 2.64 µVrms; therefore, the DR is 94.1 dB.

**Figure 16:**
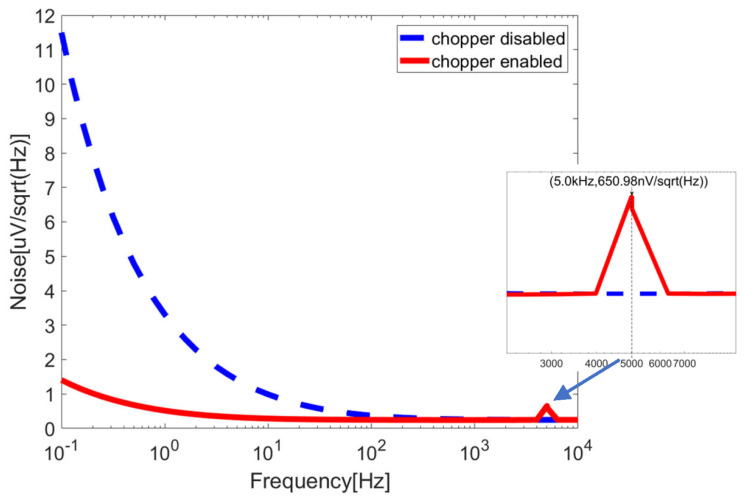
Input-referred noise spectrum of the chopper-stabilized DDA.

Figure 16 illustrates the input-referred noise spectrum of the proposed chopper-stabilized DDA (solid line), with a comparison to the noise spectrum when the chopper is disabled (dash line). Notably, chopper modulation at 5 kHz results in a reduction in low-frequency noise density by a factor of nearly 10. Additionally, the noise spectrum shows a peak at the chopping frequency, signifying the modulation of low-frequency noise into odd harmonics of the chopping frequency.

**Figure 17:**
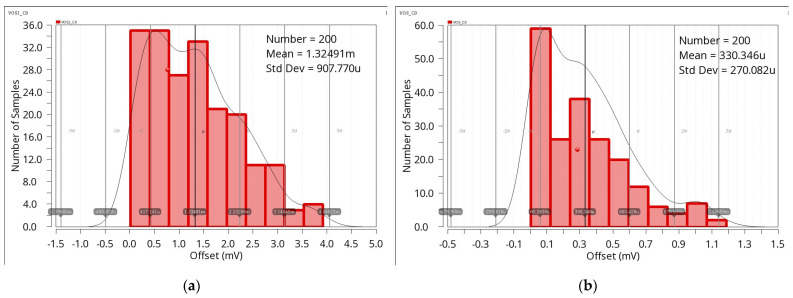
Input-referred offset: (**a**) chopper disabled, (**b**) chopper enabled.

Figure 17 shows the input-referred offset obtained by Monte Carlo simulation. Figure 17a illustrates the histogram with chopping disabled, while Figure 17b shows the histogram with chopping enabled. This comparison reveals that chopping at 5 kHz reduces the offset voltage by a factor of 3.3.

**Figure 18:**
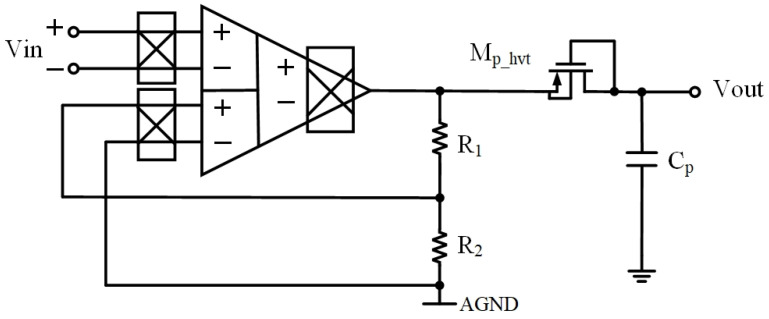
Closed-loop configuration of proposed chopper-stabilized DDA with LPF.

Figure 18 illustrates the closed-loop configuration of the chopper DDA with a low-pass filter (LPF), where AGND stands for analog ground which will be supplied by the Systems-on-Chip (SoCs). The amplifier’s dc gain is determined by (24). By setting R_2_ = 10 kΩ and R_1_ = 990 kΩ, a gain of around 40 dB can be obtained. To optimize chip area utilization, a first-order filter making use of a pseudo-resistor and a capacitor is employed [20]. The pseudo-resistor is a high-threshold PMOS transistor with gate-drain shorts, enabling the realization of large resistances in the GΩ range. Integration of the pseudo-resistor with a small capacitor results in cutoff frequencies in the range of a few hundred Hertz. The choice of high-threshold transistors serves the purpose of minimizing leakage currents, ensuring the circuit’s efficiency and reliability. The sizes and types of components are shown in Table 4.

(24) $$A_{d}=1+\frac{R_{1}}{R_{2}}$$

**Figure 19:**
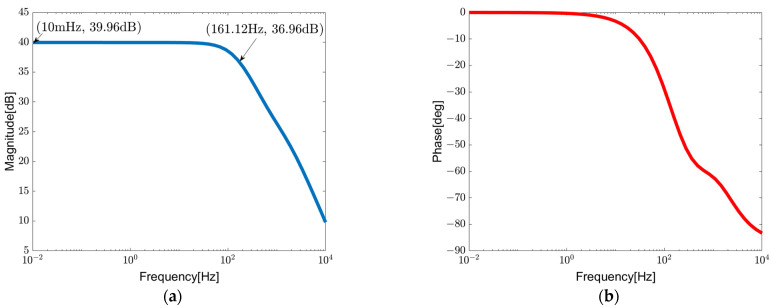
AC frequency response of closed-loop chopper-stabilized DDA with LPF: (**a**) gain, (**b**) phase.

**Figure 20:**
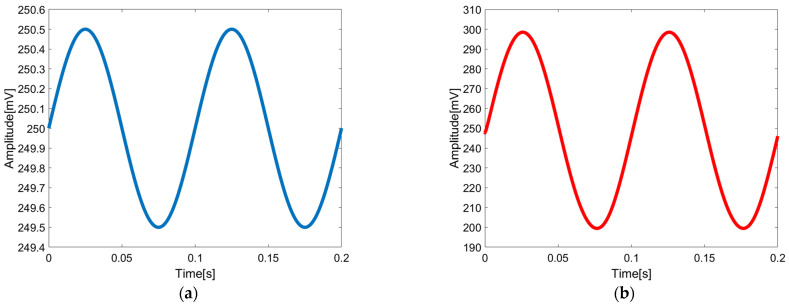
Transient response of the chopper-stabilized DDA with LPF: (**a**) input, (**b**) output.

Figure 19 and Figure 20 illustrate the AC frequency response and transient response of the closed-loop chopper DDA with LPF, respectively. It clearly reveals a 3 dB bandwidth of 161.12 Hz. Following the LPF, the input-referred noise is 2.56 μVrms.

**Figure 21:**
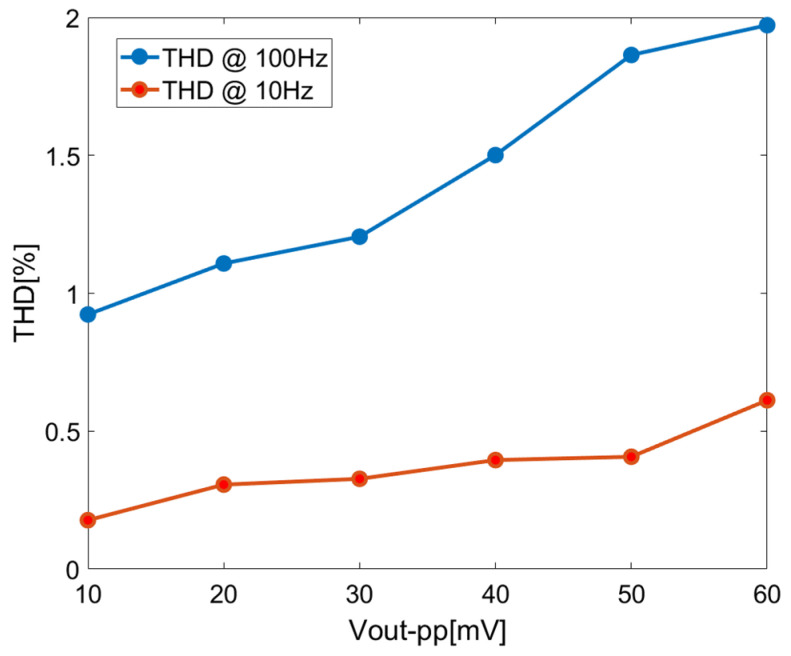
THD of closed-loop chopper DDA with LPF.

Figure 21 shows the relation between THD and the amplitude of the output signal for 10 Hz and 100 Hz input signals of the closed-loop chopper-stabilized DDA with the LPF. In this application setup, a quiescent bias of 300 mV is used in the input port to maximize the input dynamic according to the input common mode range, as shown in Figure 13. Referring to Figure 21, for THD < 2% as the limit, the amplitude of the output signal is less than 60 mVpp in rms value. Combining the output noise floor of 256 µVrms, the DR can be calculated as 38.3 dB. In general, distortion increases with the increase in closed-loop gain in the amplifier. Hence, the maximum allowable output for a particular distortion level is reduced. As such, DR is governed by the closed-loop gain factor. It is also possible to improve DR further but at the expense of increased power consumption as the tradeoff.

The results of process corners, voltage, and temperature analysis are illustrated in Table 5. Regardless of different conditions, the circuit maintains a total power consumption of under 1 µW, while achieving a gain greater than 80 dB. The data in Table 5 demonstrate the circuit’s robustness over various process variations. In general, performance can be further enhanced if more power consumption is permitted in the design.

Parasitic capacitors of layout are estimated and intentionally added at the circuit component nodes to allow evaluation of the parasitic impact on the circuit performance. These parasitic capacitors range from 40 fF to 400 fF. Table 6 presents the simulation of the chopper-stabilized DDA both with and without intentionally added parasitic capacitors. By comparing the results, it can be confirmed that the proposed chopper-stabilized DDA is minimally impacted by the parasitic effects arising from layout issues.

The tradeoff efficiency of an amplifier integrates the aspects of noise, power, and unity-gain bandwidth. To assess this, the Figure of Merit (FoM), defined as noise-power per bandwidth, is introduced. The formula is given as follows:

(25) $$FoM=P_{w}\times Input\;Noise@\;1kHz/UGB$$

where $P_{w}$ is the power consumption and UGB is the unity-gain bandwidth. The lower the FOM, the better the overall performance of the amplifier. The simulation results show that the FOM of the proposed DDA is 1.03 $\left(\left(nV/\sqrt{Hz}\right)\cdot \mu W/Hz\right)$.

Table 7 provides a comparison of the performance of the proposed chopper-stabilized DDA with other low-voltage DDAs. It is evident that the proposed circuit achieves a relatively high CMRR and PSRR. Furthermore, the incorporation of the chopper technique significantly reduces noise and input-referred offset in the circuit, making it stand out in terms of these aspects. Although the power consumption of the circuit is in the medium range, the proposed DDA exhibits the lowest value of FoM and power per bandwidth, suggesting the overall efficiency in tradeoff design when considering power, noise, and bandwidth parameters. Despite the circuit’s limited-input common-mode range, it has confirmed good performance in both gain and bandwidth. These results collectively affirm the effectiveness of the proposed circuit architecture and frequency compensation technique.

## 5. Conclusions

This paper presents a new three-stage chopper-stabilized DDA with a supply voltage of 0.5 V. It utilizes a combination of two feed-forward paths and a Type II frequency compensator to achieve a good balance between high open-loop gain, wide bandwidth, and sufficient phase margin. The integration of chopper stabilization techniques yields very favorable results in terms of offset voltage reduction and low-frequency 1/f noise suppression. The overall circuit design also offers excellent energy efficiency with very low power consumption and a very high CMRR. Extensive simulation results have validated the circuit’s robustness, demonstrating a tenfold reduction in low-frequency noise at a chopping frequency of 5 kHz. The proposed circuit will be very useful for low-voltage low-power analog signal processing applications.

## Figures and Tables

**Table 1:** Specification of the AFE for ECG monitoring.

Parameter	Size
BW	0–150 Hz
GBW	≥100 kHz
PM	>50 degree
Max Allowable Gain	100
Input ECG Signal	0.5–8 mV
Input-referred Offset	<1 mV
CMRR@10 Hz	≥100 dB
PSRR@10 Hz	>60 dB
Power Consumption	<1 μW
Slew Rate	>100 mV/uS
C_L_	50 pF

**Table 2:** Sizes and types of components of proposed chopper-stabilized DDA.

Components	Type	Size (W/L)
M_B1_	Regular NMOS	50/1 (μm/μm)
M_B2_	Regular NMOS	5/1 (μm/μm)
M_B3_, M_B4_	Low-threshold PMOS	28/1 (μm/μm)
M_B5_	Low-threshold NMOS	2.5/1 (μm/μm)
M_B6_	Low-threshold NMOS	12/1 (μm/μm)
M_1_, M_2_, M_3_, M_4_	Low-threshold NMOS	50/1 (μm/μm)
M_5_, M_6_	Low-threshold PMOS	50/1 (μm/μm)
M_7_, M_8_	Low-threshold NMOS	25/1 (μm/μm)
M_9_	Low-threshold PMOS	20/1 (μm/μm)
M_10_	Low-threshold PMOS	18.5/1 (μm/μm)
M_11_, M_12_	Low-threshold NMOS	1/1 (μm/μm)
M_13_	Low-threshold PMOS	40.5/1 (μm/μm)
M_14_	Low-threshold NMOS	26/1 (μm/μm)
M_15_	Low-threshold PMOS	8/1 (μm/μm)
M_16_	Native NMOS	70/1 (μm/μm)
M_17_	Low-threshold NMOS	1/7 (μm/μm)
M_18_	High-threshold NMOS	30/1 (μm/μm)
C_B1_, C_B2_	Capacitor	10 pF
C_m1_	Capacitor	5 pF
C_m2_	Capacitor	250 fF
C_L_	Capacitor	50 pF
R_B_	Resistor	4.5 MΩ
R_m1_	Resistor	1.2 MΩ
R_L_	Resistor	300 kΩ

**Table 3:** Sizes of components in clock booster.

Component	Size	Component	Size
M_1_, M_2_	3/1 (μm/μm)	C1	5 pF
M_3_, M_5_, M_7_	6/1 (μm/μm)	C2	1 pF
M_4_, M_6_, M_8_	3/1 (μm/μm)		

**Table 4:** Sizes and types of components of closed-loop chopper-stabilized DDA.

Components	Type	Size (W/L)
M_p_hvt_	High-threshold PMOS	100/1 (μm/μm)
C_P_	Capacitor	1 pF
R_1_	Resistor	990 kΩ
R_2_	Resistor	10 kΩ

**Table 5:** Simulated main performance parameters in unity-gain configuration over process voltage and temperature variations.

Parameter (T = 27 °C, V_DD_ = 0.5 V)	TT	SS	FF
Gain, (dB)	88.87	91.87	81.09
UGB, (kHz)	170.46	113.49	239.68
PM, (deg)	63.98	53.24	66.28
GM, (dB)	18.58	20.50	15.64
PSRR, (dB)	78.56	80.42	76.84
CMRR, (dB)	100.81	96.58	101.79
SR+, (V/ms)	150.00	66.92	216.49
SR−, (V/ms)	54.54	34.12	386.67
Input Noise, @1 kHz (nV/sqrt (Hz))	245.45	295.23	194.94
Power Consumption, (μW)	0.72	0.61	0.95
C_L_	50 pF	50 pF	50 pF
**Parameter** **(TT corner, T = 27 °C)**	**0.45 V**	**0.5 V**	**0.55 V**
Gain, (dB)	83.03	88.87	88.17
UGB, (kHz)	136.76	170.46	170.34
PM, (deg)	52.20	63.98	63.9
GM, (dB)	17.70	18.58	17.97
PSRR, (dB)	56.00	78.56	78.5
CMRR, (dB)	78.12	100.81	100.78
SR+, (V/ms)	22.36	150.00	251.26
SR−, (V/ms)	29.62	54.54	309.33
Input Noise, @1 kHz (nV/sqrt (Hz))	269.03	245.45	245.08
Power Consumption, (μW)	0.59	0.72	0.99
C_L_	50 pF	50 pF	50 pF
**Parameter** **(TT corner, V_DD_ = 0.5 V)**	**−20 °C**	**27 °C**	**80 °C**
Gain, (dB)	93.16	88.87	80.02
UGB, (kHz)	90.24	170.46	425.39
PM, (deg)	59.10	63.98	52.02.
GM, (dB)	14.36	18.58	22.14
PSRR, (dB)	76.00	78.56	92.08
CMRR, (dB)	95.89	100.81	85.16
SR+, (V/ms)	22.17	150.00	181.25
SR−, (V/ms)	23.15	54.54	226.89
Input Noise, @1 kHz (nV/sqrt (Hz))	221.89	245.45	148.27
Power Consumption, (μW)	0.53	0.72	1.00
C_L_	50 pF	50 pF	50 pF

**Table 6:** Simulated main performance parameters with and without intentionally added parasitic capacitors.

Parameter	Without Intentionally Added Parasitic Capacitors	With Intentionally Added Parasitic Capacitors
Gain, (dB)	88.87	88.10
UGB, (kHz)	170.46	170.72
PM, (deg)	63.98	63.65
GM, (dB)	18.58	17.88
PSRR, (dB)	78.56	78. 87
CMRR, (dB)	100.81	101.17
Input Noise, @1 kHz (nV/sqrt (Hz))	245.45	245.58
Power Consumption, (μW)	0.72	0.72
C_L_	50 pF	50 pF

**Table 7:** Performance comparison with other reported works.

	Units	This Work	[28] 2022	[29] 2019	[30] 2018	[31] 2018
Process	μm	0.04	0.18	0.18	0.18	0.065
Supply Voltage	V	0.5	0.5	0.5	0.5	1
Power consumption	μW	0.72	0.313	1.23	0.59	1.12
ICMR	mV	200	500	500	500	-
Open-loop DC gain	dB	89	95	62	64.7	104.4
UGB	kHz	170.46	12.82	56.4	33.3	-
Power/Bandwidth	μW/kHz	0.004	0.024	0.022	0.018	-
Phase Margin	(°)	63.98	55.7	54	58	-
CMRR @ DC	dB	101	60	58	78	124
PSRR @ DC	dB	79	66	60	62	88
Input Noise	nV/sqrt(Hz)	245	880	-	578	-
Input-referred Offset	mV	0.264	6.14	3.4	4.75	-
FOM	$(nV/\sqrt{Hz})\cdot \mu W/Hz$	1.03	21.49	-	10.24	-
Average SR	V/ms	150.00	16.25	51.9	93	-
SR+	V/ms	54.54	15.81	74	152	-
SR-	V/ms	102.27	16.69	29.9	34	-
C_L_	pF	50	15	30	20	-

## Data Availability

Data are contained within the article.
